# The Diagnostic and Predictive Significance of Immune-Related Genes and Immune Characteristics in the Occurrence and Progression of IgA Nephropathy

**DOI:** 10.1155/2022/9284204

**Published:** 2022-04-28

**Authors:** Jian-Bo Qing, Wen-Zhu Song, Chang-Qun Li, Ya-Feng Li

**Affiliations:** ^1^The Fifth Clinical Medical College of Shanxi Medical University, Taiyuan, Shanxi 030001, China; ^2^School of Public Health, Shanxi Medical University, Taiyuan, Shanxi 030001, China; ^3^Department of Nephrology, Shanxi Provincial People's Hospital (Fifth Hospital) of Shanxi Medical University, Taiyuan, Shanxi 030012, China; ^4^Core Laboratory, Shanxi Provincial People's Hospital (Fifth Hospital) of Shanxi Medical University, Taiyuan, Shanxi 030012, China; ^5^Shanxi Provincial Key Laboratory of Kidney Disease, Shanxi Provincial People's Hospital (Fifth Hospital) of Shanxi Medical University, Taiyuan, Shanxi 030012, China; ^6^Academy of Microbial Ecology, Shanxi Medical University, Taiyuan, Shanxi 030000, China

## Abstract

**Objective:**

To investigate the potential diagnostic and predictive significance of immune-related genes in IgA nephropathy (IgAN) and discover the abnormal glomerular inflammation in IgAN.

**Methods:**

GSE116626 was used as a training set to identify different immune-related genes (DIRGs) and establish machine learning models for the diagnosis of IgAN; then, a nomogram model was generated based on GSE116626, and GSE115857 was used as a test set to evaluate its clinical value. Short Time-Series Expression Miner (STEM) analysis was also performed to explore the changing trend of DIRGs with the progression of IgAN lesions. GSE141344 was used with DIRGs to establish the ceRNA network associated with IgAN progression. Finally, ssGSEA analysis was performed on the GSE141295 dataset to discover the abnormal inflammation in IgAN.

**Results:**

Machine learning (ML) performed excellently in diagnosing IgAN using six DIRGs. A nomogram model was constructed to predict IgAN based on the six DIRGs. Three trends related to IgAN lesions were identified using STEM analysis. A ceRNA network associated with IgAN progression which contained 8 miRNAs, 14 lncRNAs, and 3 mRNAs was established. A higher macrophage ratio and lower CD4+ T cell ratio in IgAN compared to controls were observed, and the correlation between macrophages and monocytes in the glomeruli of IgAN patients was inverse compared to controls.

**Conclusion:**

This study reveals the diagnostic and predictive significance of DIRGs in IgAN and finds that the imbalance between macrophages and CD4+ immune cells may be an important pathomechanism of IgAN. These results provide potential directions for the treatment and prevention of IgAN.

## 1. Introduction

IgA nephropathy (IgAN) is inflammatory nephropathy characterized by IgA deposition in the mesangial area of the glomeruli [1]. It represents the most common primary glomerular disease globally [2], and its prevalence varies geographically, more frequent in Asian populations (45 million people/year in Japan) than in Caucasians (31 million people/year in France) [3]. Also, IgAN owns a higher incidence in young adults [4], with 20-40% of patients subject to end-stage renal disease within 10-20 years [5]. Worse still, IgAN is a lifelong disease with severe signs and symptoms closely associated with poor prognosis, posing a heavy mental and financial burden to patients [6].

It is crucial to make an early diagnosis of IgAN since delayed diagnosis would contribute to a poor prognosis [7]. Nowadays, pathological biopsy represents the golden standard in IgAN diagnosis, but the results are changeable with the stage of the disease [8]. Besides, the pathological results are sometimes uncertain, making IgAN diagnosis and evaluation tricky. As such, an effective and reliable diagnostic method for IgAN is all the more important. Currently, it has been documented that the occurrence and development of IgA are closely related to genetic [9, 10]. As the microarray technique plus bioinformatics advances, it is a good approach to utilize genes to make a diagnosis and risk assessment for IgAN. Marker genes can not only help us diagnose, but also help us explore the molecular mechanism, signaling pathway, and pathological progress of IgAN.

This study is aimed at exploring the role of genes in the occurrence and progression of IgAN, using integrated gene expression profiling data downloaded from the Gene Expression Omnibus (GEO) database, and at further identifying immune-related genes as diagnostic biomarkers for IgAN patients, which may contribute to the diagnosis and treatment of IgAN. Additionally, abnormal immune infiltration in the glomerulus was studied in patients with IgAN. These results will contribute to better diagnosis, prevention, and treatment of IgAN. In this study, we first identified genes for constructing models and then further explored the gene network related to IgAN progression. Finally, we analyzed the immune infiltration of IgAN glomerulus.

## 2. Materials and Methods

### 2.1. Data Collection

#### 2.1.1. GEO Dataset Download and Process

We used the keyword “IgA nephropathy” to search IgAN gene expression profiles in the GEO database. The following steps to obtain dataset: first, screen datasets for constructing a diagnostic model, whose organization used for sequencing must be the kidney and have cases and controls. Second, search the noncoding RNA profiling dataset used for exploring the ceRNA network of IgAN. Third, as IgAN features glomerular disease and the proportion of differentially expressed genes detected by RNA-SEQ was higher than that by CHIP, the RNA-SEQ dataset of glomerular tissue was selected. Fourth, the datasets must be published within the last five years. Finally, the GEO datasets numbered GSE116626, GSE115857, GSE141344, and GSE141295 were selected. The summary of those four GEO datasets is shown in Table 1.

With more evenly distributed data and more detailed records of the IgAN lesions, GSE116626 was employed for the training set and used for differential expression analysis, and the characteristics of samples are shown in Table 2. Moreover, GSE115857 was utilized as the test set. We then performed log2 transformed for gene expression profiling and gene symbol conversion; in addition, quality control was performed on each dataset to improve the efficiency of subsequent analysis.

#### 2.1.2. Immune-Related Gene Download

**Figure 1:**
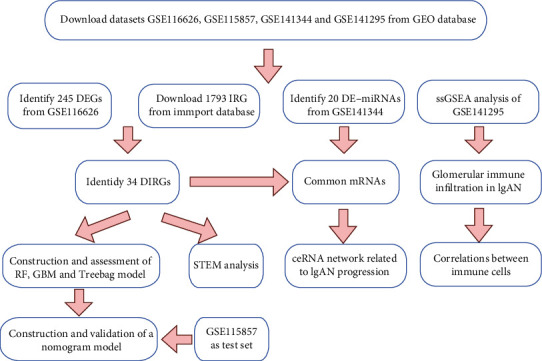
Workflow of the research. Abbreviations are defined as follows: Gene Expression Omnibus database (GEO), differentially expressed gene (DEG), immune-related gene (IRG), different immune-related gene (DIRG), differentially expressed miRNA (DE-miRNA), random forest (RF), Gradient Boosting Machine (GBM), Short Time-Series Expression Miner (STEM).

We downloaded immune-related genes (IRGs) data from the import database (https://www.immport.org/shared/) [11]. After eliminating duplicate genes, we finally obtained 1793 immune-related genes (Supplementary Table 1). The schematic of the research is shown in Figure 1.

### 2.2. Differential Expression Analysis and Enrichment Analysis

Differentially expressed genes (DEGs) between IgAN and healthy control in GSE116626 were achieved by the package “limma” [12] in R software with a criterion of *P* < 0.05 and ∣log_2_fold change | >1. Afterwards, a comparison was made between 245 DEGs and 1793 immune-related genes (IRGs) to obtain different immune-related genes (DIRGs). Furthermore, for deep insight into the gene functions, the package “clusterProfiler” [13] in R was employed for the Gene Ontology (GO) and Kyoto Encyclopedia of Genes and Genomes (KEGG) analysis. Finally, we visualized the results by bubble map in R software.

### 2.3. Construction and Assessment of RF, GBM, and Treebag Model

There are 83 samples in our test set, which contains 52 IgAN patients, 22 non-IgAN patients (non-IgAN GN), and 7 healthy people; although artificial neural networks (ANN) or deep neural networks (DNN) are effective learning models in the prediction process, they are poorly interpretable and require more samples [14]; thus, machine learning (ML) will be more advantageous in this study. Firstly, as elastic net [15] has less prediction error than lasso and ridge regression in our test, it was employed for gene screening in 34 DIRGs using the “glmnet” package in R; the alpha = 1 and lamda = 6 (CV.Glmnet function automatically produces the most appropriate value). Besides, we divided the samples of GSE116626 into three types, random forest (RF), Gradient Boosting Machine (GBM), and treebag model as they could achieve the optimal effect in the ML of the three classification samples. After which, we used GSE116626, with IgAN as the response variable, and DIRGs as explanatory variables, to establish RF, GBM, and treebag model. What is more, we employed the package “DALEX” in R software to analyze the above three models and plot the residual distribution.

### 2.4. Construction and Validation of a Nomogram Model for IgAN Diagnosis

A nomogram model was established to predict the occurrence of IgAN using the “rms” package after three ML models verified the accuracy of the DIRGs. “Points” indicates the score of the corresponding factors below, and “total points” indicates the sum of all the scores of factors above used to estimate the risk of IgAN. In addition, a calibration curve was used to evaluate the predictive performance of the nomogram model, while decision curve analysis and clinical impact curve were further used to evaluate the clinical value of the model.

### 2.5. Short Time-Series Expression Miner (STEM) Analysis

We used STEM software [16] to cluster the 34 DIRGs in 4 different lesions of IgAN patients and healthy controls in the GSE116626 datasets; *α* < 0.05 was considered statistically significant clustering. This method can help us to simulate and estimate the spatiotemporal variation of DIRGs in IgAN. The significantly clustered genes showed a gradual up- or downregulation trend with the change of IgAN lesion.

### 2.6. Identification of miRNA Related to IgAN Progression

The package “DESeq2” [17] in R was used to seek for differently expressed miRNAs (DE-miRNAs) between IgAN progression (IgAp) and IgAN nonprogression (IgAnp) with *P* < 0.05 and ∣log_2_fold change | >1 criterion in GSE141344. Samples were classified as IgANnp if the serum creatinine had changed by <10% over the 10 years since diagnosis, while samples whose serum creatinine had at least doubled or the patient had developed end-stage renal disease(ESRD) in the same time period were classified as IgANp. Afterwards, mRNAs targeted by MiRNAs with experimental support were identified using miRDB [18], miRWalk [19], and miRanda [20] databases. To make our results more convincing, we first incorporated the common genes from the three databases and then compared them with the 34 DIRGs for the final target mRNAs. Also, starBase v 3.0 [21] was used to predict the lncRNAs of miRNAs with final target RNAs, with search parameters set at “CLIP-Data ≥ 5”. Finally, we built the ceRNA network based on the above data, and the ggalluvial (version 0.9.1) package [22] was used to visualize the network.

### 2.7. Immune Signatures of the Glomeruli in IgAN

The ssGSEA was applied to explore the different infiltrations of immune cells in GSE141295 using the R package “GSVA” [23]. Afterwards, we compared the ratio of 24 immune cells between the IgAN and control groups and the correlation change of 10 main immune cells between the two groups.

## 3. Results

### 3.1. Differential Expression Analysis and Enrichment Analysis

**Figure 2:**
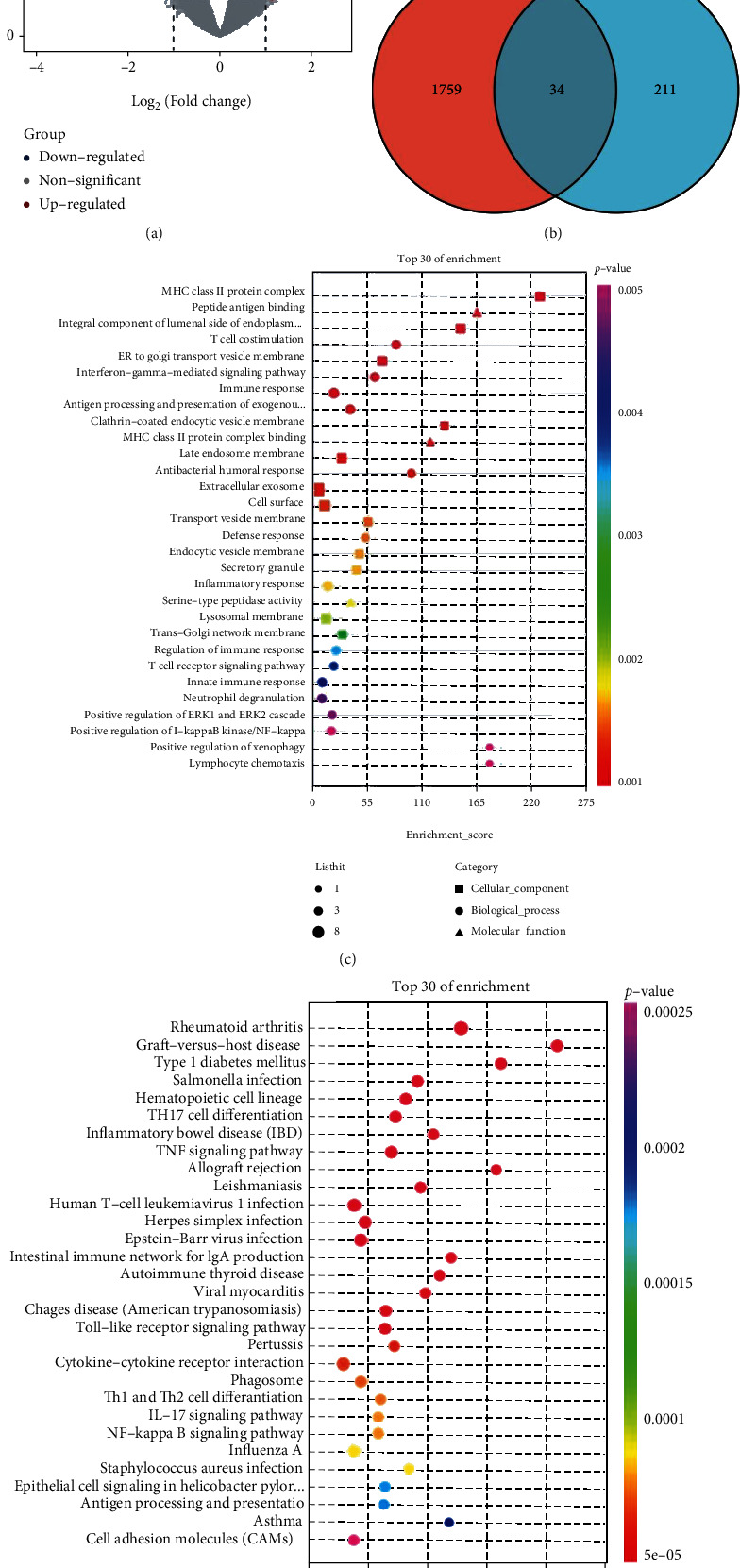
Differential expression analysis and enrichment analysis in GSE116626. (a) Volcano map of all DEGs of GSE116626: 245 DEGs were identified with *P* < 0.05 and ∣log_2_fold change | >1, including 160 upregulated and 85 downregulated genes. (b) Venn diagram of DEGs and IRGs: 34 DIRGs were identified. (c) Top 30 GO functional enrichment of 34 DIRGs. Biological process (BP, circle), cellular component (CC, square), and molecular function (MF, triangle) analysis results of 34 DIRGs. (d) Top 30 KEGG functional enrichment of 34 DIRGs. The size of the graph represents the number of genes, and the *x*-axis represents the enrichment score.

245 DEGs between IgAN and control groups in GSE116626 were identified with *P* < 0.05 and ∣log_2_fold change | >1, including 160 upregulated and 85 downregulated genes (Figure 2(a)). Also, 34 DIRGs were obtained from 245 DEGs and 1793 IRGs (Figure 2(b)), whose enriched pathways and functions were further understood by GO and KEGG enrichment analysis (Supplementary Table 2 and 3). GO analysis showed that the 34 DIRGs were mainly associated with MHC class II protein complex, peptide antigen binding, and interferon-gamma-associated pathway, interferon-gamma-mediated signaling, and pathway positive regulation of I-kappaB kinase/NF-kappaB signaling (Figure 2(c)). Meanwhile, KEGG analysis showed that these 34 DIRGs were closely related to autoimmune diseases, differentiation of T cell helper, and intestinal immune network for IgA production. It suggested that these 34 DIRGs were bound up with autoimmune diseases (Figure 2(d)).

### 3.2. Construction and Assessment of RF, GBM, and Treebag Model

**Figure 3:**
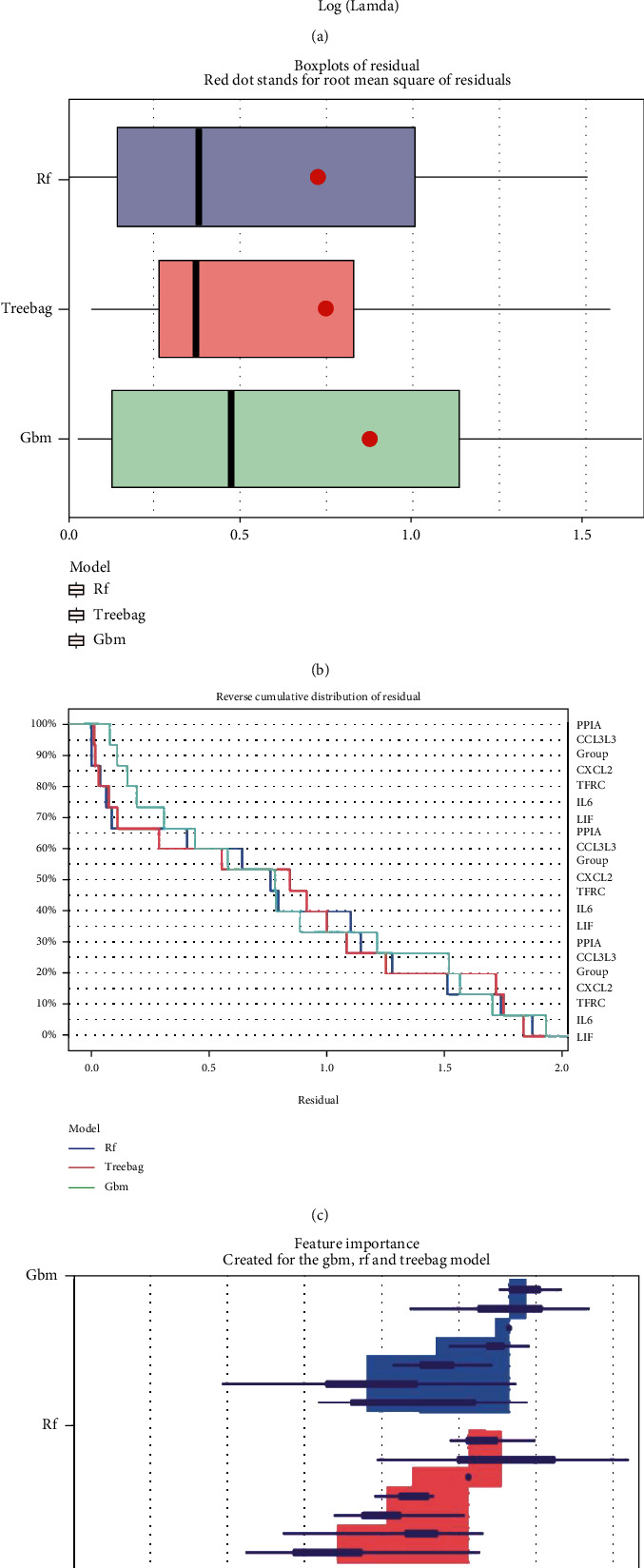
Construction and assessment of RF, GBM, and treebag model. (a) Elastic net of 34 DIRGs in GSE116626. The main parameters are alpha = 1 and lamda = 6 (CV.Glmnet function automatically produces the most appropriate value.). (b) Cumulative residual distribution map of the sample. (c) Boxplots of the residuals of the sample. Red dot stands for root mean square of residuals. The residual distributions were very close in the three models. (d) Importance of the variables in RF, GBM, and treebag model. The six DIRGs have different importance in three models.

The elastic net revealed six genes (PPIA, CCL3L3, CXCL2, TFRC, IL6, and LIF) (Figure 3(a)), based on alpha = 1 and lamda = 6, which were taken into the ML, namely, RF, GBM, and treebag models. There were 100 trees in the RF models; besides, the final values used for the GBM model were n.trees = 50, interaction.depth = 2, shrinkage = 0.1, and n.minobsinnode = 10; what is more, the bagging regression trees of the treebag model have 25 bootstrap replications. The three models were then analyzed using the explanatory features of the “DALEX” package in R, and the residual distributions were plotted to obtain the optimal model at the bottom of the test set (Figures 3(b) and 3(c)); additionally, Figure 3(d) shows us the importance of six genes. The results showed that the three machine learning models enjoy a good performance; ML was successfully used to identify six genes that could be applied to clinical diagnosis and prediction.

### 3.3. Construction and Assessment of a Nomogram Model for IgAN Diagnosis

**Figure 4:**
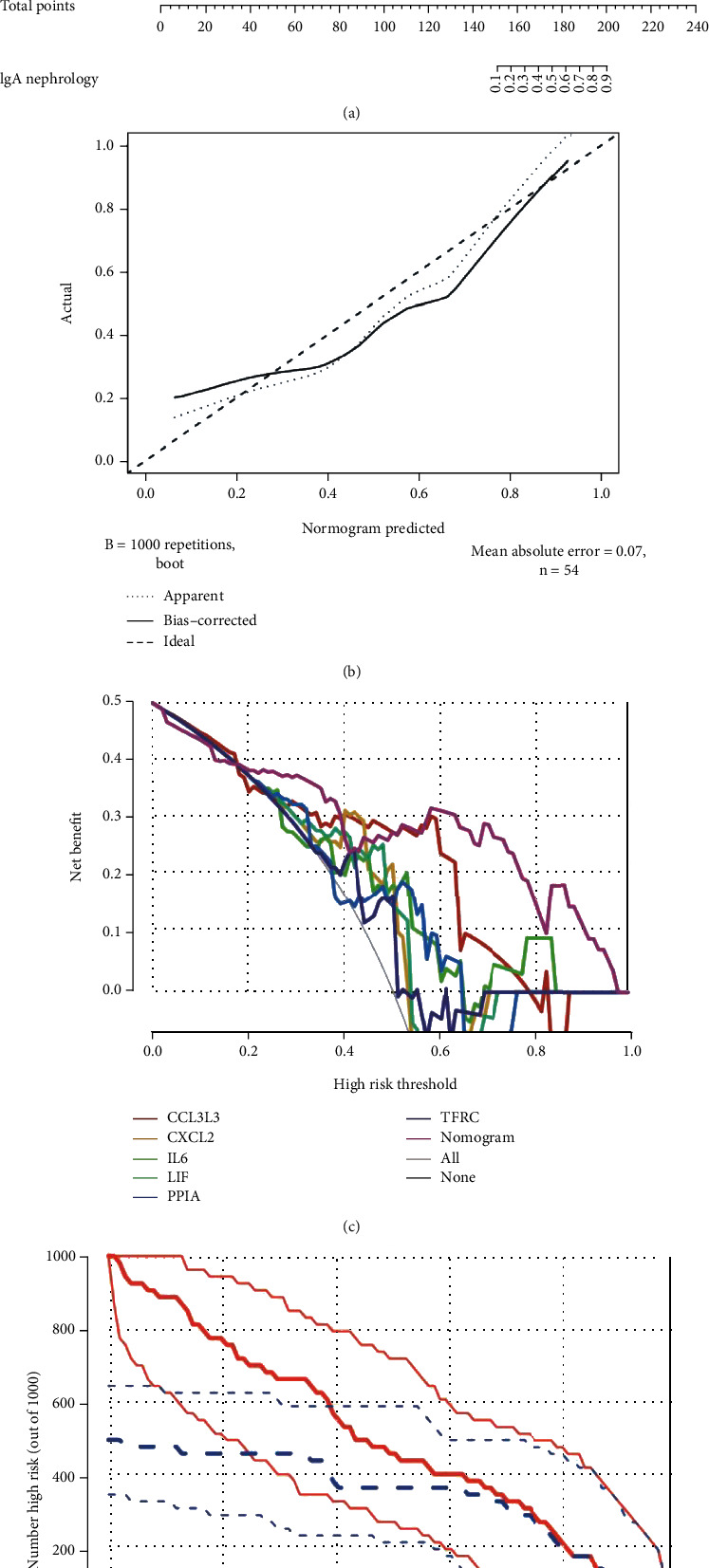
Construction and validation of a nomogram model for IgAN diagnosis. (a) Nomogram model for IgAN diagnosis, based on the 6 DIRGs (PPIA, CCL3L3, CXCL2, TFRC, IL6, and LIF). (b) Calibration curve to evaluate the nomogram model. The actual IgAN risk and the predicted risk are very close. (c) DCA curve to assess the nomogram model. Different colors represent different combinations of variables, and the nomogram model has better benefits than other models in most risk thresholds. (d) The clinical impact curve based on the DCA curve to evaluate the nomogram model, and the nomogram model has a better clinical effect when the risk threshold exceeded 0.6.

The “rms” package was used to establish the nomogram model for IgAN diagnosis based on the 6 DIRGs (PPIA, CCL3L3, CXCL2, TFRC, IL6, and LIF) (Figure 4(a)). The calibration curve which was used to evaluate the predictive power of the nomogram model indicated that the actual IgAN risk and the predicted risk are very close, suggesting that the nomogram model owns high accuracy to predict IgAN (Figure 4(b)). Furthermore, decision curve analysis (DCA) indicated that the “nomogram” curve had the highest benefits in most risk threshold ranges, especially if the risk threshold exceeds 0.6, indicating that the nomogram model boosts good predictions (Figure 4(c)), and the clinical impact curve on the ground of the DCA curve was conducted to evaluate the clinical effects of the nomogram model more visually (Figure 4(d)), which indicated that the prediction of the nomogram model is very close to the actual event when the risk threshold exceeds 0.6. The results indicated that the nomogram model owns great potential in predicting the risk of IgAN.

### 3.4. Short Time-Series Expression Miner (STEM) Analysis

**Figure 5:**
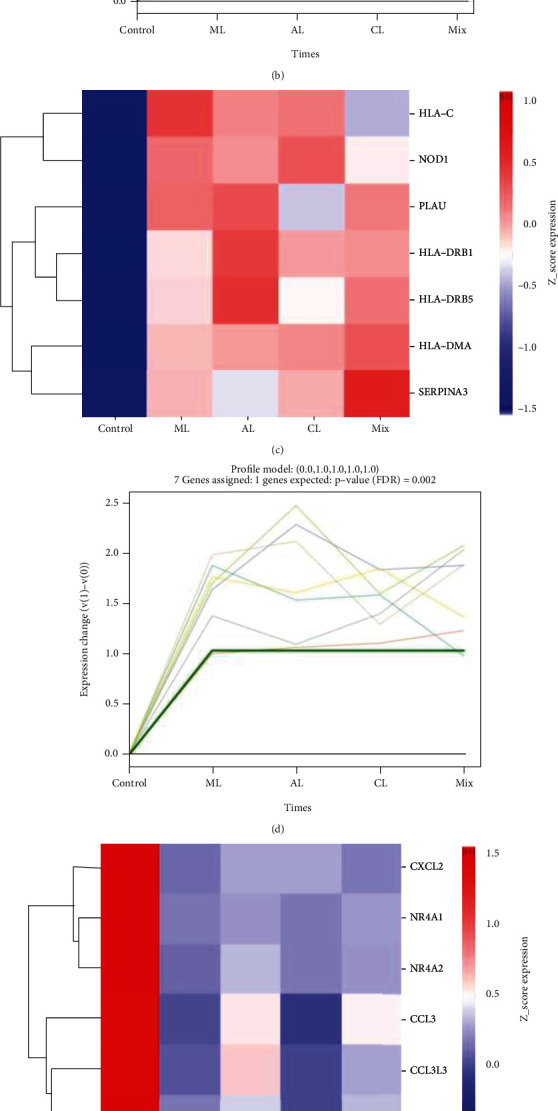
Short Time-Series Expression Miner (STEM) analysis. (a) Heatmap of six gene expressions in trend 1, including 6 DIRGs, ITGAL, TUBB3, ADRB2, SLP1, CCL19, and CTSG. (b) Line chart of six gene expressions in trend 1, FDR = 0.00017. (c) Heatmap of seven gene expressions in trend 2, including 7 DIRGs, HLA-C, NOD1, PLAU, HLA-DRB1, HLA-DRB5, HLA-DMA, and SERPINA3. (d) Line chart of seven gene expressions in trend 2, FDR = 0.002. (e) Heatmap of six gene expressions in trend 3, including 6 DIRGs, CXCL2, NR4A1, NR4A2, CCL3, CCL3L3, and IL6. (f) Line chart of six gene expressions in trend 3, FDR = 0.0085.

STEM software was used to determine the alteration in 34 DIRGs during the progression from healthy controls to different IgAN lesions. These genes were divided into three significant clusters (Figures 5(a)–5(f)). Among them, ITGAL, TUBB3, ADRB2, SLP1, CCL9, and CTSG showed a trend of gradual increase with different IgAN stages, with a peak at the mixed lesion, suggesting that these six genes may be associated with different pathological types of IgAN alterations. These genes in the three significant clusters may represent biomarkers of IgAN lesions.

### 3.5. Identification of ceRNA Related to IgAN Progression

**Figure 6:**
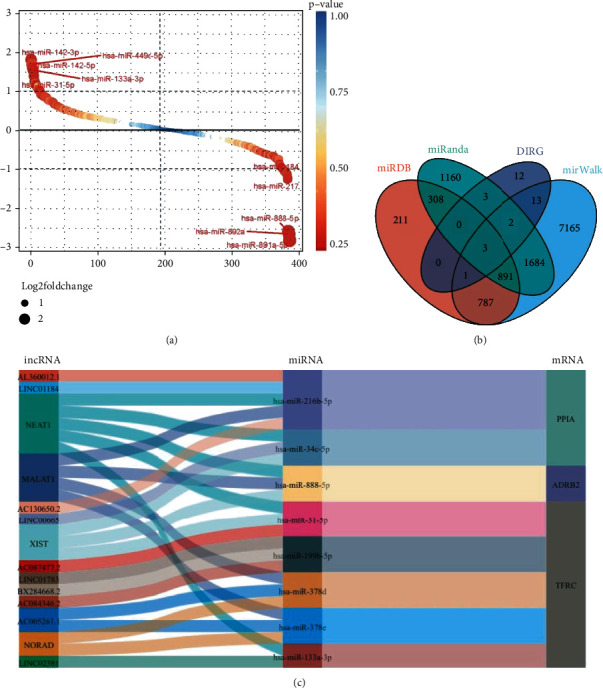
Identification of ceRNA related to IgAN progression. (a) Gene rank of DE-miRNAs in GSE141344. 10 upregulated and 10 downregulated DE-miRNAs were identified using the package “DESq2” at *P* < 0.05 and ∣log_2_fold change | >1. (b) Venn diagram of 34 DIRGs and the data from miRDB, miWALK, and miRanda databases. There were three common targeted DIRGs (PPIA, ADRB2, and TFRC) in these three databases. (c) ceRNA network related to IgAN progression, including 8 miRNAs, 14 lncRNAs, and 3 mRNAs.

We first detected 10 upregulated and 10 downregulated DE-miRNAs in IgAp using the package “DESq2” at *P* < 0.05 and ∣log_2_fold change | >1 (Figure 6(a)), after which miRDB, miRWalk, and miRanda databases were employed for target predictions of these 20 DE-miRNAs (Supplementary Tables 4–6). Surprisingly, PPIA, ADRB2, and TFRC were obtained in comparison to 34 DIRGs of IgAN in the GSE116626 (Figure 6(b)). Also, lncRNAs, which target the DE-miRNA related to PPIA, ADRB2, and TFRC, were explored by the starBase v 3.0 database (Supplementary Table 7). Finally, we established a ceRNA network with 8 miRNAs, 14 lncRNAs, and 3 mRNAs (Figure 6(c)).

### 3.6. Immune Signatures of the Glomeruli in IgAN

**Figure 7:**
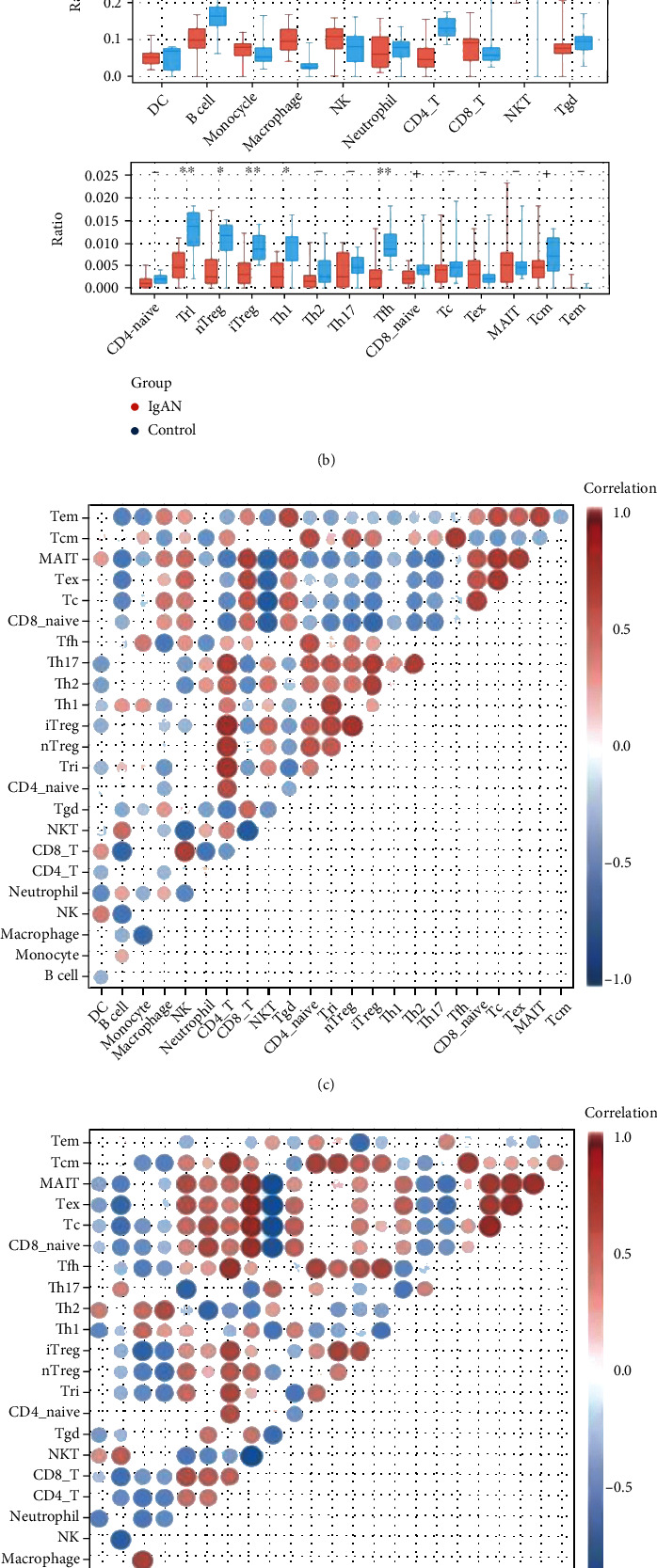
Immune signatures of the glomeruli in IgAN. (a) Heatmap of 24 immune cells calculated with ssGSEA in IgAN and healthy controls. (b) Boxplot of 24 immune cells in IgAN and healthy controls. Macrophages and NKT cells were significantly increased, while B cells, CD4+ T cells, Tr1, Treg, and Th1 cells were significantly decreased in IgAN. (c) Correlation heatmap of 10 main immune cells in IgAN. (d) Correlation heatmap of 10 main immune cells in healthy controls. (e) Line regression of monocytes and macrophages in IgAN and healthy controls. The proportion of monocytes and macrophages was negatively correlated in IgAN (*R*^2^ = 0.52, *P* = 0.003) but negatively correlated in the control group (*R*^2^ = 0.59, *P* = 0.009).

ssGSEA analysis was first applied to explore the abnormal glomerular immune infiltration in IgAN. Afterwards, 24 types of immune cells in IgAN patients and controls were generated and compared, as shown in Figure 7(a). Clearly, IgAN patients showed a significant increase in macrophages and NKT cells, and a significant decrease in B cells, CD4+ T cells, Tr1, Treg, and Th1 cells in the glomeruli (Figures 7(b) and 7(c)). Besides, by comparing correlations between 10 main immune cells, correlation changes were detected between IgAN patients (Figure 7(d)) and controls (Figure 7(e)), among which changes between macrophages and monocytes are the most obvious (Figure 7(f)). As such, the occurrence of IgAN is closely related to abnormal immune cell infiltration, and the correlation change between immune cells may be implicated in the occurrence and progression of IgAN.

## 4. Discussion

With the increase in kidney biopsy, the underestimated prevalence of IgAN will be revealed [24]. Many studies have shown that IgAN involves not only young adults but also the elderly [25]. The existing diagnosis owns some limitations for early diagnosis of IgAN, and the pathogenesis of IgAN is extremely complicated, involving biochemical, immunological, and genetic [26], which brings challenges to the prevention and therapy of IgAN. As such, new biomarkers and diagnostic models are needed urgently. Recent genetic findings have confirmed the strong role of genetics and suggested geographical and ethnic differences in IgAN susceptibility, among which Asians are more inclined to genetic risks [10].

### 4.1. The Function of the DIRGs

The enrichment analysis indicated that 34 DIRGs not only represent an important player in IgAN but also serve as a bridge between IgAN and other immune diseases. GO analysis showed that the interferon-*γ*-mediated signaling pathway is closely associated with IgAN, and the production of IFN-*γ* in IgAN was positively correlated with total IgA levels [27]. Meanwhile, altered ERK1/2 expression, which has been shown to highly influence mesangial cell proliferation [28], positively regulates the ERK1 and ERK2 cascades that may be involved in IgAN development. Positive regulation of the I-kappaB kinase/NF-kappaB signaling pathway would promote IgAN [29], and many studies have shown that drugs could treat IgAN by inhibiting this pathway [30]. Regulation of related gene expression may have a positive effect on IgAN.

What is more, KEGG enrichment analysis showed that T cell helper (Th) differentiation, TNF signaling pathway, and IgA-generating intestinal immune network are involved in the pathogenesis of IgAN. IgAN is regulated by Th [31]. Interestingly, it has been shown that the use of antitumor necrosis factor- (TNF-) alpha agents in rheumatoid arthritis (RA) could lead to the development of IgAN and diabetes [32, 33], which may be interrelated because of similar abnormal gene expression and thus have some signaling pathways running through many diseases, such as inflammatory bowel disease (IBD) [34]. Notably, intestinal immunity is a major player in IgAN [35], and it has become an important foothold for IgAN treatment and exploration, but the mechanisms involved are complex [36]. The discovery of DIRGs may help us to further uncover their underlying pathogenesis. The results of our enrichment analysis for 34 DIRGs are parallel to the current study, and exploring the pathogenesis from a genetic perspective may provide new ideas for treatment of IgAN.

### 4.2. The Models Used for Diagnosis and Prediction of IgAN

We first employed elastic net to screen six genes (PPIA, CCL3L3, CXCL2, TFRC, IL6, and LIF) from 34 DIRGs, which were used for model construction, including RF, treebag, and GBM models. It turned out that three models performed excellently, the residuals of the sample in three models are small and close, which indicated that the six DIRGs were used to diagnose and predict IgAN with credible precision and accuracy. We managed to narrow down the number of IRGs to six, which is of great significance for clinical practice. Afterwards, the six genes were utilized for the construction of the nomogram model, which owns great prediction performance from the nomogram model at a high-risk threshold from 0 to 1; the calibration curve, DCA curve, and clinical impact curve indicated that the nomogram model boosts good predictions. With growing renal biopsies in patients with kidney disease, patients can benefit from the nomogram model.

### 4.3. STEM Analysis and ceRNA Network for Exploring the Progression of IgAN

The possible space-time variations of the 34 DIRGs in different IgAN lesions were discovered through STEM analysis, which could help find potential biomarkers related to disease progression. One of the most interesting changes was in ITGAL TUBB3, ADRB2, SLP1, and CCL19 CTSG, with slightly lower expression of the minimal lesion and active lesion and significantly higher than the chronic and mixed lesion. ITGAL is involved in various immune phenomena including leukocyte-endothelial cell interactions, cytotoxic T cell-mediated killing, and antibody-dependent killing by granulocytes and monocytes [37]. CCL19 plays an important role in the trafficking of T cells in the thymus and T cell and B cell migration to secondary lymphoid organs [38]; CTSG regulates inflammation, activating matrix metalloproteinases (MMPs) and coagulation [39]. Besides, TUBB3 [40], ADRB2 [41], and SLP1 [42] are also involved in the immune response, such as cytokine network and antibacterial response. Furthermore, HLA-DRB1, HLA-DRB5, and HLA-DMA are broadly expressed in different lesions of IgAN, with the strongest correlation between HLA and IgAN [43]. Studies have shown that HLA-DB1 may be associated with IgAN progression [44]. These genes may serve as biomarkers for predicting IgAN lesion and progression as IgA progression is closely related to changes in gene expression.

Moreover, we further analyzed miRNAs associated with IgAN progression and generated a ceRNA network of 8 miRNAs, 14 lncRNAs, and 3 mRNAs based on miRDB miWALK and miRNADA and starBase v 3.0. miRNAs have been associated with immune and pathological changes in the kidney through regulating gene expression [45]. The TFRC gene involved in ferroptosis and some other important pathogenesis has been linked to a variety of diseases [46] and may be linked to IgAN susceptibility; meanwhile, NEAT1, MALAT1, and XIST which have the most degree with miRNA in the ceRNA network were identified; NEAT1 may affect IgAN by regulating the TLR2/NF-*κ*B signaling pathway [47]. Besides, research once showed that IgAN mesangial cells displayed increased expression of MALAT1 [48], and XIST could mediate inflammatory response via NF-*κ*B/NLRP3 inflammasome pathway, which may be the potential pathomechanism by which it affects the IgAN progression.

### 4.4. The Changes of Immune Landscape in the Glomeruli of IgAN

IgAN is the most common primary glomerulonephritis worldwide and is characterized by abnormal immune cell infiltration [49]. ssGAEA analysis was applied to explore the immunological signatures of IgAN in glomerular biopsy tissue. Deposits of immune complexes containing galactosylated IgA1 activate mesangial cells, leading to an overproduction of local cytokines, chemokines, and complement [50]. Macrophages, which are significantly higher in IgAN, could amplify the effects of inflammatory factors and participate in and contribute to the process of mesangial hyperplasia and glomerulosclerosis [51]. The prolonged macrophage infiltration may promote mesangial cell proliferation or the development of extracapillary lesions that eventually lead to the progression of IgAN [52]. Besides, activation of NKT cells is associated with the production of mucosal IgA [53] and an increased ratio of NKT is involved in IgAN immunopathology [54].

Additionally, the ratio of CD4+ T cells is significantly reduced in IgAN patients. Its reduction is mainly manifested in regulatory T cell (Treg) and helper T cell (Th). Since the reduction in Treg is associated with abnormal immune function in patients with IgAN [55], it is of great significance for the prognosis of IgAN. Thus, increasing the ratio of Treg may protect the renal function of patients [56, 57]. Meanwhile, the reduction of Th1 and Tfh in glomeruli is an integral part of IgAN and they play an active role in maintaining a normal immune response to IgAN and stopping its progression [58, 59]. Although B cell infiltration also increased in IgAN, it owns an insignificant effect. Finally, by analyzing the correlation between immune cells, we found that the correlation between macrophages and monocytes in the glomeruli of IgAN patients was inverse. The results may indicate a significant increase in differentiation of monocytes into macrophages, which would emerge as a potential mechanism and therapeutic target for IgAN.

Although lots of work have been done, there are some limitations in our study. Firstly, the clinical information of the samples is not rich enough, such as renal function, urine protein, and blood pressure; more risk factors make more accurate model. Secondly, the expression level of DIRGs and the immune landscape of IgAN may need further verification. We will enrich and verify our conclusions on real clinical cases in the following study.

## 5. Conclusion

The diagnostic model of IgAN is constructed using IRGs in our study, and we find that the imbalance between macrophages and CD4+ immune cells may be an important pathomechanism of IgAN. These results provide potential directions for the treatment and prevention of IgAN. Our study draws on a wide range of data and methods to reach rich conclusions that will be translated into clinical practice in the future, and we will explore new directions for IgAN treatment based on our findings in this study.

## Figures and Tables

**Table 1:** The summary of those four GEO datasets.

GSE number	Samples	Organization	Purpose
GSE116626	52 IgAN patients, 22 non-IgAN GN, and 7 controls	Kidney	Training set
GSE115857	55 IgAN patients, 7 controls	Kidney	Test set
GSE141344	6 IgANp patients and 6 IgANnp patients	Kidney	ceRNA network
GSE141295	14 IgAN patients and 10 controls	Glomeruli	ssGSEA

Abbreviation: IgAN: IgA nephropathy; non-IgAN GN: non-IgA nephropathy glomerulonephritis; IgANp: IgA nephropathy with progression; IgANp: IgA nephropathy with no progression.

**Table 2:** The characteristics of samples in the GSE116626 dataset.

Group	Number	MEST-C classification
Minimal lesion IgAN	22	(M0,1; E0; S0,1; T0; C0)
Active lesion IgAN	8	(M0,1; S0,1; T0; E1 and/or C,1,2)
Chronic lesion IgAN	12	(M0,1; E0; S0,1; T1,2; C0)
Mixed lesion IgAN	10	(M0,1; E0,1; S0,1; T1,2; C0,1,2)
Non-IgAN GN	7	—
Healthy control	22	—

Abbreviation: IgAN: IgA nephropathy; non-IgAN GN: non-IgA nephropathy glomerulonephritis.

## Data Availability

The raw data supporting the conclusions of this article will be made available in the GEO database. All data generated or analyzed during this study are included in this article and its supplementary information files.

## Supplementary Materials

Supplementary Materials Supplementary Table 1: the immune-related genes downloaded from the import database (https://www.immport.org/shared/). Supplementary Table 2: all data of GO enrichment analysis. Supplementary Table 3: all data of KEGG enrichment analysis. Supplementary Table 4: the target predictions of these 20 DE-miRNAs in the miRDB database. Supplementary Table 5: the target predictions of these 20 DE-miRNAs in the miRWalk database. Supplementary Table 6: the target predictions of these 20 DE-miRNAs in the miRanda database. Supplementary Table 7: the lncRNAs which target the DE-miRNA related to PPIA, ADRB2, and TFRC in the starBase v 3.0 database.
